# Radioiodine refractoriness score: A multivariable prediction model for postoperative radioiodine‐refractory differentiated thyroid carcinomas

**DOI:** 10.1002/cam4.1794

**Published:** 2018-09-27

**Authors:** Genpeng Li, Jianyong Lei, Linlin Song, Ke Jiang, Tao Wei, Zhihui Li, Rixiang Gong, Jingqiang Zhu

**Affiliations:** ^1^ Thyroid and Parathyroid Surgery Center West China Hospital of Sichuan University Chengdu China

**Keywords:** differentiated thyroid carcinoma, radioiodine refractoriness, risk factors, scoring system

## Abstract

**Objective:**

The purpose of the present study was to evaluate the clinical features of patients with radioiodine refractory (RAIR) differentiated thyroid carcinoma (DTC) and establish an effective risk score for postoperative radioiodine refractoriness.

**Subjects and methods:**

Data were retrospectively collected from 5163 patients admitted to our center after thyroid surgery. Radioiodine refractoriness was defined according to criteria used in the 2015 American Thyroid Association guidelines. The scoring system was established by independent risk factors identified by univariate and multivariate analyses. The optimal index points for predicting the prevalence of radioiodine refractoriness and the model discriminatory power were assessed by receiver operating characteristic (ROC) curves.

**Results:**

One hundred and twelve (2.2%) patients developed RAIR DTC. Smoking, tumor type (follicular thyroid cancer), extrathyroid extension, lymph node metastasis number (≥4), lymph node metastasis rate (≥53%), and pN stage (N1) were highly positively correlated with the prevalence of RAIR DTC. The cutoff value of seven points was found to be the best for predicting the prevalence of RAIR DTC, and the scoring system presented better discrimination than other single independent predictors.

**Conclusions:**

Based on our multivariable prediction model, patients with ≥7 index points may need to undergo more active surveillance or aggressive treatment due to the high risk of RAIR DTC.

## 1 INTRODUCTION

Differentiated thyroid carcinomas (DTCs), including papillary and follicular types based on histopathological criteria, account for approximately 90% of thyroid malignancies.^1^ The standard primary treatments for DTCs mainly include surgery, radioactive iodine (RAI) therapy, and thyroid‐stimulating hormone (TSH) suppression therapy.^2^ Although most DTC patients show no evidence of disease with appropriate early therapeutic modalities, recurrence develops in 20%‐40% of patients.^3^ During tumor progression, RAI therapy is occasionally the first line of treatment when the lesion becomes too advanced to resect surgically or metastasizes to distant organs.^4^ However, up to 5 percent of thyroid cancer metastases can lose their ability to concentrate iodine and are referred to as radioiodine refractory (RAIR); this phenomenon is responsible for a large number of deaths attributed to thyroid cancer.^5^

Due to the unfavorable prognosis of DTC that are RAIR, mechanisms underlying the dedifferentiation process have been studied and comprise the lost expression of thyroid‐specific genes, such as the human sodium‐iodine symporter; these changes are frequently caused by genetic aberrations activating the BRAF, RET, and phosphatidylinositol 3‐kinase‐AKT pathways.^6^, ^7^, ^8^, ^9^ Meanwhile, multiple strategies have been investigated for their potential to improve the iodide uptake of dedifferentiated thyroid carcinoma, such as the application of retinoic acid, histone modification agents, inhibitors of mammalian target of rapamycin (mTOR), and multikinase inhibitors (MKIs).^2^, ^3^, ^10^ However, factors associated with a high risk of RAIR cancer have rarely been mentioned in the literature, and these factors could not only help the comprehension of the natural history of RAIR cancer but also facilitate the optimization of patient management.

In the present study, we focused on investigating the association of different clinical parameters with RAIR cancer to identify independent predictors and established an effective multivariable prediction model to evaluate the risk of RAIR cancer.

## 2 SUBJECTS AND METHODS

From January 2012 to December 2016, the medical records of 5163 patients who were treated for thyroid diseases at the West China Hospital of Sichuan University were reviewed. The inclusion criteria were DTC patients who underwent total thyroidectomy and neck dissection including prophylactic central lymph node dissection, therapeutic central lymph node dissection, and/or therapeutic lateral lymph node dissection, were treated with RAI (100 mci), were diagnosed with RAIR cancer at 18‐80 years of age, and had adequate available medical data. Central lymph node dissection was extended superiorly to the hyoid bone, inferiorly to the innominate vein, laterally to the carotid sheaths, and dorsally to the prevertebral fascia. Lateral lymph node dissection was performed using a modified radical operation that involved complete removal of level II through IV lateral cervical lymph node. Level I and V dissection was not performed if there was no clinical evidence of metastases. In our study, individuals with other pathological types of thyroid cancer, lobectomy, RAI therapy (not 100 mci), and incomplete medical records were excluded. RAIR cancer was detected by CT, MRI, and ^131^I whole‐body scans. RAIR cancer was defined according to the criteria used in the 2015 American Thyroid Association guidelines, as follows: (a) metastatic disease that does not take up RAI at the time of the first ^131^I treatment; (b) ability to take up RAI lost after previous evidence of uptake; (c) RAI uptake retained in some lesions but not in others; or (d) metastatic disease that progresses despite substantial uptake of RAI.^11^ Based on the inclusion and exclusion criteria, 112 subjects were eligible for this retrospective analysis. The control group for comparison consisted of 224 randomly selected patients who underwent postoperative RAI therapy (100 mci, with the same level of TSH before RAI as the experimental group) and did not exhibit RAIR cancer and those cases did not match with group of RAIR to avoid ignoring certain risk factors. The data on the subjects’ clinical features, such as age at diagnosis, gender, body mass index (BMI), blood pressure status, prediabetes and diabetes, smoking, alcohol consumption, and autoimmune thyroid disease, were extracted from the electronic medical records. The follow‐up period ranged from 16 to 68 months (median, 32 months), and to date, 11 patients have died of thyroid carcinoma. The institutional review board approved our study design and protocol, and patient approval or informed consent was required for our review of the patients’ medical records.

Statistical analysis was performed using SPSS software, version 20.0 (SPSS, Chicago, IL, USA). Continuous and categorical data are expressed as the mean ± SD and the number, respectively, and the differences were compared and analyzed using a Student's sample *t* test, chi‐squared test, or Fisher's exact test. The Kaplan‐Meier method and log‐rank test were used to analyze time‐dependent variables. Univariate analyses were used to identify factors associated with RAIR cancer. Multivariate logistic regression was performed for all variables that were significant in the univariate analysis. Odds ratios (ORs) and 95% relative confidence intervals (CIs) were calculated to determine the relevance of all potential predictors. According to multiple logistic regression analyses, features that were independent factors were assigned different points based on the OR to develop a scoring system. Receiver operating characteristic (ROC) curves were used to determine the optimal cutoff values. Box plots and pyramid figures were used to compare average scores and display scoring distributions. The best point with a high sensitivity and low false‐negative rate (1‐specificity) was identified. A *P* value <0.05 was considered indicative of statistical significance.

## 3 RESULTS

### 3.1 RAIR population

**Figure 1:**
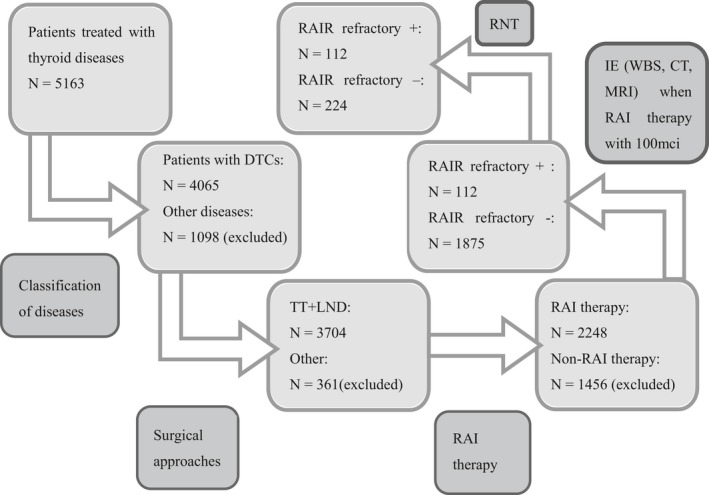
Flowchart of the population. Of the 5163 patients treated for thyroid disease in our center, we isolated 112 RAIR patients between January 2012 and December 2016. The control group for comparison consisted of 224 randomly selected patients who underwent postoperative RAI therapy and did not exhibit RAIR cancer. The dose of RAI and value of TSH before RAI in two groups were no differences. DTC, differentiated thyroid carcinoma; IE, imaging examination; LND, lymph node dissection; RAIR, radioiodine refractory; RNT, random number table; TT, total thyroidectomy

**Figure 2:**
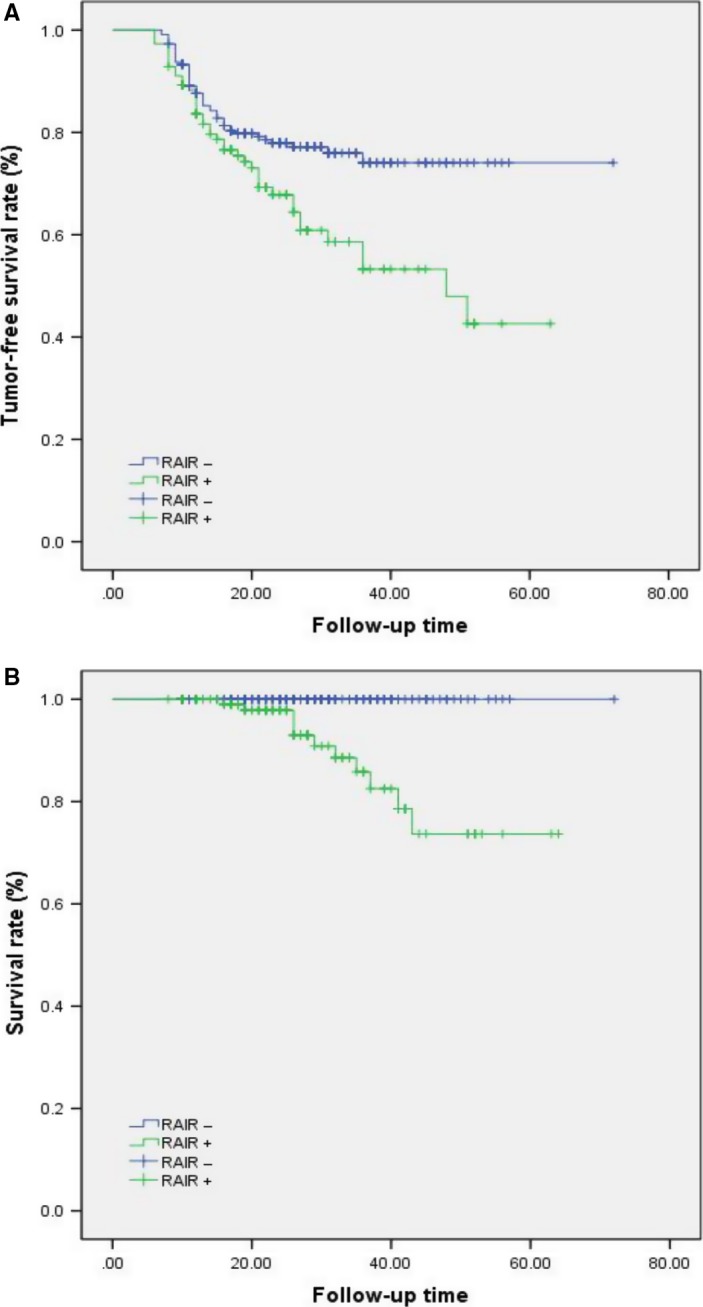
**A,** The tumor‐free survival rate of radioiodine refractory (RAIR) patients was significantly lower than that of non‐RAIR patients (*P* = 0.002). **B,** The overall survival of RAIR patients was significantly lower than that of non‐RAIR patients (*P* = 0.011)

From January 2012 to December 2016, of the 5163 patients initially treated for thyroid disease, 4065 patients were diagnosed with DTC, and 3704 patients underwent total thyroidectomy in our department. RAI therapy (30‐200 mCi) after surgery was administered to 2248 patients. To improve comparability, of the 2248 patients whose imaging studies were reviewed when individuals treated with most common dose in first time (100 mCi), 112 (103 papillary thyroid cancers, nine follicular thyroid cancers) met the study criteria and were included in the present analysis. Meanwhile, the control group for comparison consisted of randomly selected patients treated with 100 mCi in first time as well among patients who underwent RAI therapy postoperation and did not exhibit RAIR cancer and there were no differences of value of TSH before RAI in RAIR+ and RAIR− groups (Figure 1). During the follow‐up period, deaths were related to thyroid cancer in 11 cases. As shown in Figure 2A,B, the tumor‐free survival and overall survival rates were significantly decreased for patients with RAIR cancer.

### 3.2 Variables of patients affecting the development of RAIR cancer on univariate analysis

**Figure 3:**
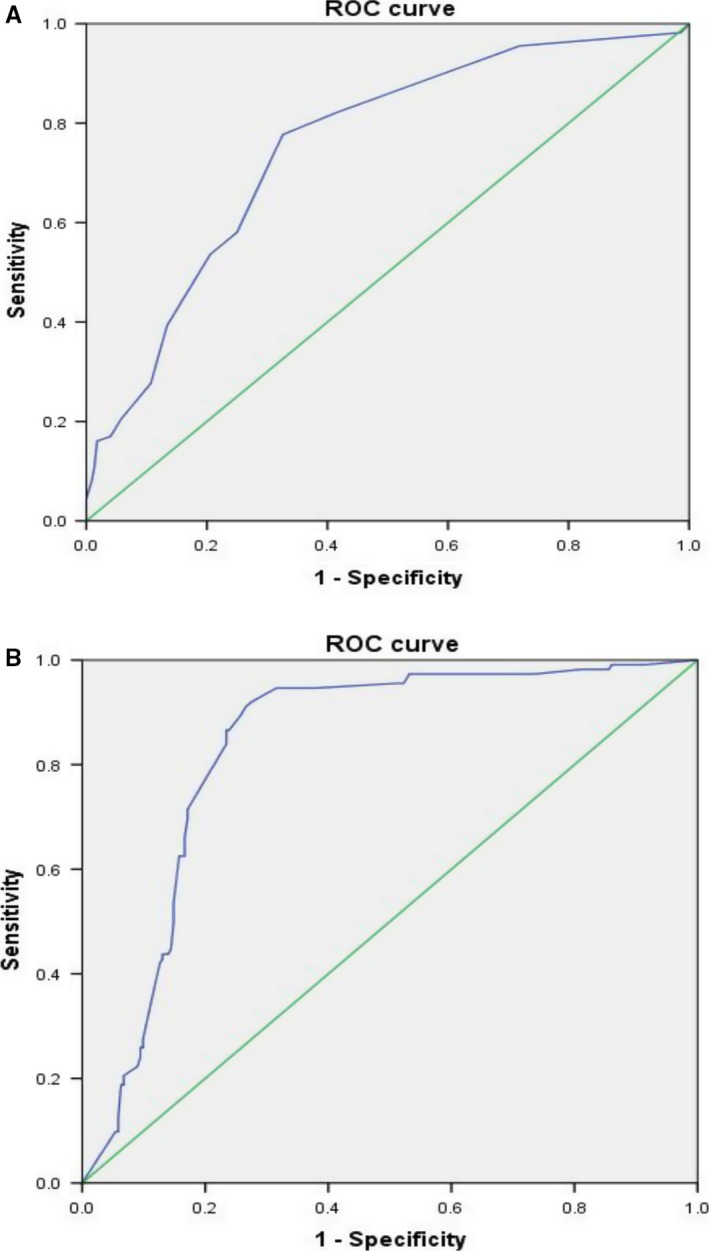
Receiver operating characteristic (ROC) curve to predict the prevalence of radioiodine refractory cancer. **A,** The optimal metastatic lymph node number was 4, and the area under the ROC curve (AUC) was 0.753. The sensitivity and specificity were 77.7% and 67.4%, respectively. **B**, The optimal metastatic lymph node rate was 53%, and the area under the ROC curve (AUC) was 0.833. The sensitivity and specificity were 86.6% and 76.6%, respectively

The baseline demographics and tumor characteristics are listed and compared between the two groups in Table 1. We found that the number of lymph node metastases and the rate of lymph node metastasis were significant differences between two groups but the largest diameter of metastasized lymph nodes in RAIR group and control group was at the same level in preliminary data. Thus, ROC curves were used to determine the optimal cutoff values to predict the prevalence of RAIR cancer in terms of the number of lymph node metastases and the rate of lymph node metastasis (determined by histopathology), which were 4 (AUC = 0.753) and 53% (AUC = 0.833), respectively (Figure 3A,B). Comparing RAIR and non‐RAIR patients by univariate analysis, the following 12 factors significantly increased the risk of RAIR cancer: age at diagnosis ≥55 years (*P* = 0.017); BMI ≥24 kg/m^2^ (*P* = 0.004); smoking (*P* = 0.001); primary tumor size >10 mm (*P* = 0.035); primary tumor size >20 mm (*P* = 0.020); tumor type (*P* = 0.024); extrathyroid extension (*P* = 0.032); lymph node metastasis number (*P* = 0.000); lymph node metastasis rate (*P* = 0.000); pT classification (*P* = 0.030); pN stage (*P* = 0.001); and pTNM stage (*P* = 0.011).

**Table 1:** Comparison of the baseline demographics and tumor features of radioiodine refractory (RAIR+) and RAIR− patients

	RAIR+ group	RAIR− group	*P* value
	112	224	
Age at diagnosis (mean ±SD, years)	41.9 ± 13.9	40.9 ± 11.2	0.431
≥55/<55	22/90	23/201	0.017*
Gender (male/female)	28/84	64/160	0.519
Height (cm)	162.3 ± 7.4	161.7 ± 6.7	0.494
Weight (kg)	62.2 ± 11.7	60.3 ± 9.7	0.149
BMI (mean ± SD, kg/m^2^)	23.5 ± 3.3	23.0 ± 3.0	0.196
≥18.5	106	218	0.212
≥24	61	85	0.004*
≥28	4	21	0.056
≥32	1	4	0.525
Blood group (A/B/AB/O)	50/39/14/39	92/71/23/86	0.532
Hypertension (yes/no)	7/105	20/204	0.395
Prediabetes or diabetes (yes/no)	3/109	5/219	0.8
Smoking (yes/no)	27/85	24/200	0.001*
Alcohol consumption (yes/no)	25/87	36/188	0.161
Nodular goiter (yes/no)	64/48	133/91	0.818
Autoimmune thyroid disease (yes/no)	34/78	66/158	0.866
Graves's disease (yes/no)	5/107	8/216	0.689
Primary tumor size (mean ± SD, mm)	15.8 ± 10.9	13.3 ± 8.2	0.019*
>10 mm	74	121	0.035*
>20 mm	25	28	0.020*
>40 mm	5	3	0.077
Tumor location (isthmus/left/right lobe)	3/50/59	5/101/118	0.938
Tumor location (upper/middle or lower)	33/79	62/162	0.732
Tumor type (FTC/PTC)	9/103	3/221	0.002*
Extrathyroid extension (yes/no)	33/79	36/188	0.009*
Multifocality (yes/no)	37/75	62/162	0.31
Lymph node metastasis number (≥4/<4)	88/24	70/154	0.000*
Lymph node metastasis rate (≥53%/<53%)	76/36	38/186	0.000*
pT classification (T1‐T2/T3‐T4)	31/81	89/135	0.030*
pN stage (N0 or N1a/N1b)	1/55/56	16/164/44	0.000*
pTNM stage (I‐II/III‐IV, AJCC version 8)	90/22	201/23	0.011*

BMI, body mass index; FTC, follicular thyroid cancer; P, pathological; PTC, papillary thyroid cancer; SD, standard deviation.

a Statistically significant difference.

### 3.3 Independent predictors associated with the prevalence of RAIR on multivariate analysis

The OR for the prevalence of RAIR cancer was identified by a logistic regression model based on the above‐mentioned factors in the univariate analysis. Controlling for 12 variables, age at diagnosis ≥55 years (OR, 1.078; 95% CI, 0.762‐1.589), BMI ≥24 kg/m^2^ (OR, 0.549; 95% CI, 0.283‐1.065), primary tumor size >10 mm (OR, 1.374; 95% CI, 0.696‐2.710), primary tumor size >20 mm (OR, 0.612; 95% CI, 0.244‐1.534), pT classification (OR, 0.889; 95% CI, 0.439‐1.802), and pTNM stage (OR, 1.714; 95% CI, 0.808‐1.707), did not show a significant difference. However, smoking (OR, 2.304; 95% CI, 1.055‐5.032), tumor type (OR, 8.104; 95% CI, 1.319‐49.811), extrathyroid extension (OR, 2.403; 95% CI, 1.080‐5.343), lymph node metastasis number (OR, 3.399; 95% CI, 1.735‐6.659), lymph node metastasis rate (OR, 6.418; 95% CI, 1.955‐23.060), and pN stage (OR, 3.185; 95% CI, 1.615‐6.281) showed highly independent associations with the occurrence of RAIR cancer. These results are shown in Table 2.

**Table 2:** Multivariate analyses of factors contributing to radioiodine refractory+ status

Variable	Odds ratio	95% CI	*P* value
Age at diagnosis ≥55 years	1.078	0.762‐1.589	0.432
BMI ≥24 kg/m^2^	0.549	0.283‐1.065	0.076
Smoking	2.304	1.055‐5.032	0.036*
Primary tumor size >10 mm	1.374	0.696‐2.710	0.36
Primary tumor size >20 mm	0.612	0.244‐1.534	0.295
Tumor type (FTC)	8.104	1.319‐49.811	0.024*
Extrathyroid extension	2.403	1.080‐5.343	0.032*
Lymph node metastasis number (≥4/<4)	3.399	1.735‐6.659	0.000*
Lymph node metastasis rate (≥53%/<53%)	6.418	1.955‐23.060	0.000*
pT classification (T1‐T2/T3‐T4)	0.889	0.439‐1.802	0.744
PN stage (N0 or N1a/N1b)	3.185	1.615‐6.281	0.001*
pTNM stage (I‐II/III‐IV, AJCC version 8)	1.714	0.808‐1.707	0.399

BMI, body mass index; FTC, follicular thyroid cancer; p, pathological

a Statistically significant difference.

### 3.4 Scoring system for predicting the prevalence of RAIR cancer

**Figure 4:**
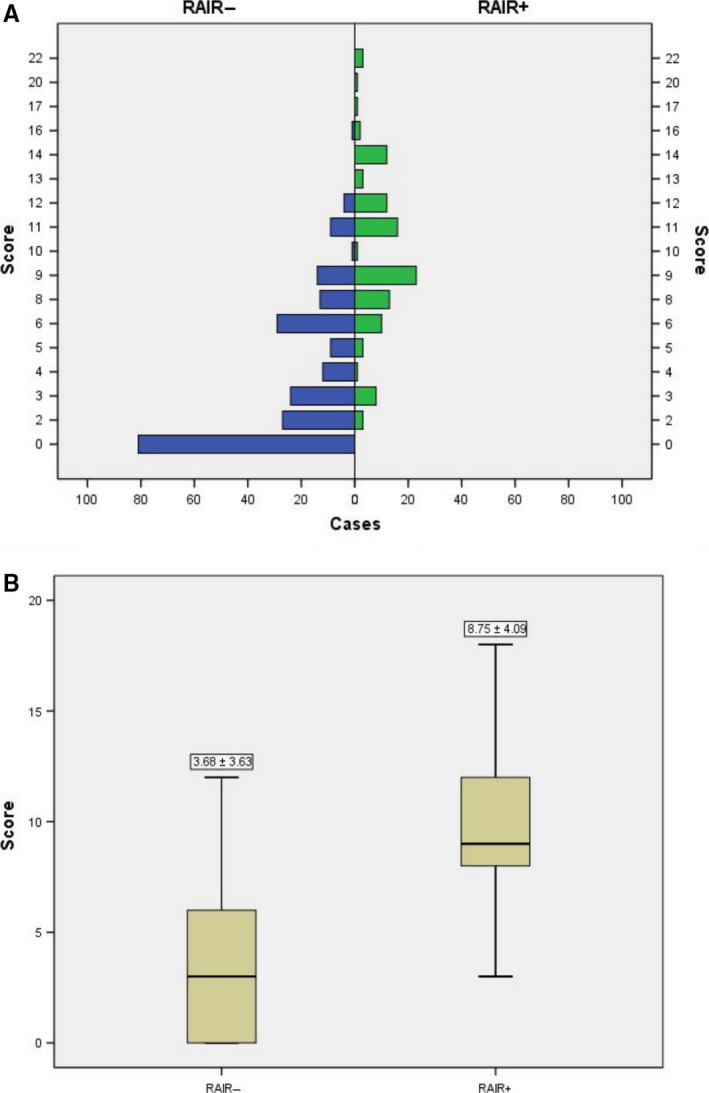
**A,** The distribution of cases in the two groups according to the scoring system. **B,** The mean index points in the radioiodine refractory (RAIR+) and RAIR− groups according to the scoring system. Significant differences were found between the two groups (*P* < 0.001)

**Figure 5:**
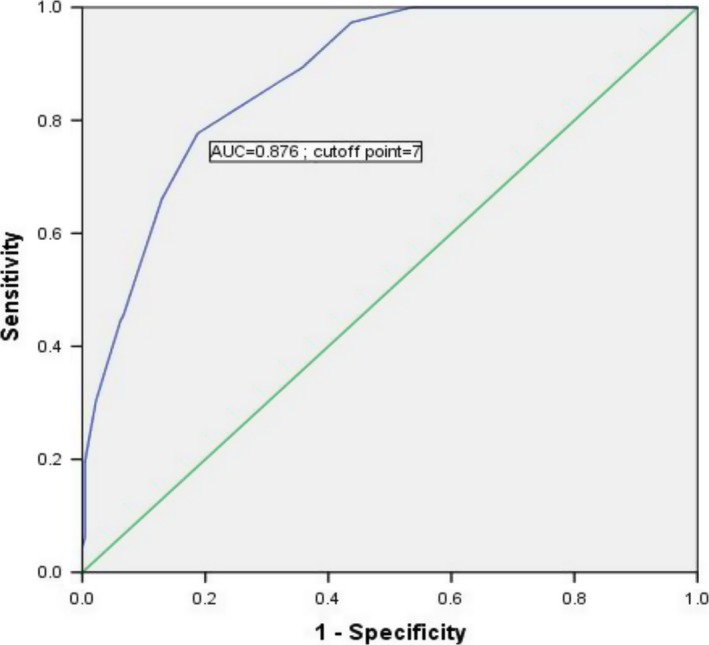
Receiver operating characteristic (ROC) curve to identify the optimal cutoff value for predicting the prevalence of RAIR cancer based on the scoring system. The area under the ROC curve (AUC) was 0.876, and a cutoff value of 7 points was found to be the best for distinguishing between RAIR and non‐RAIR patients. The sensitivity and specificity were 77.7% and 81.2%, respectively

**Figure 6:**
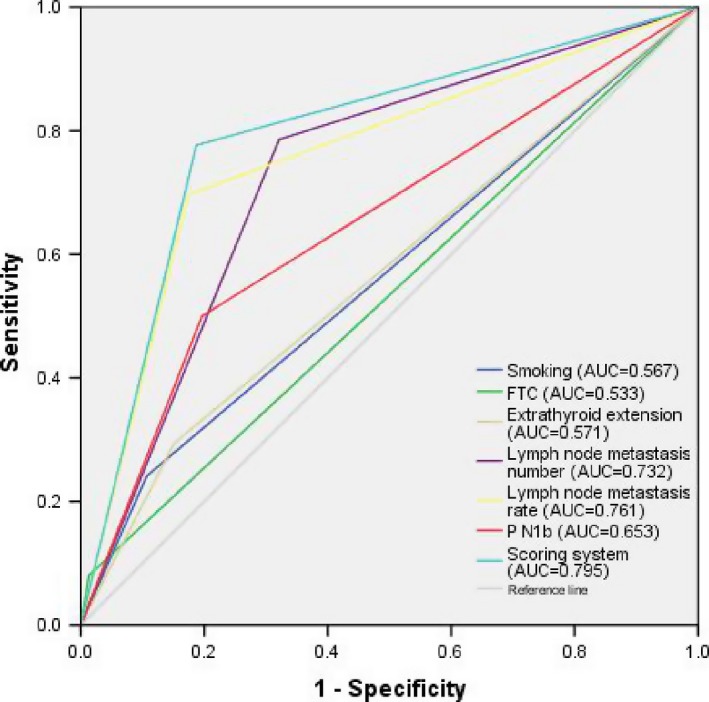
The areas under the receiver operating characteristic (ROC) curve (AUCs) for other single independent predictors (largest AUC = 0.761) showed lower discrimination power than that of the scoring system (AUC = 0.795)

Based on the multivariate logistic regression analysis, features that were associated with the development of RAIR cancer were assigned different points according to the OR value, and the evaluated cutoff points for each characteristic are shown in Table 3. The sum of the points was evaluated to distinguish between RAIR +and RAIR ‐ subjects. The score distributions are presented in Figure 4A. The number of cases decreased with increasing score in the non‐RAIR group and increased followed by a decrease with increasing score in the RAIR group. The mean index scores were significantly different, at 8.75 ± 4.09 in the RAIR group and 3.68 ± 3.63 in the non‐RAIR group, as shown in Figure 4B. Finally, as determined using ROC curves, a cutoff value of seven was found to be the best score for predicting the prevalence of RAIR cancer, with an area under the ROC curve (AUC) of 0.876 (Figure 5). The sensitivity, specificity, and Youden index of this scoring system were 77.7%, 81.2%, and 0.589, respectively. Compared with other single independent predictors, the scoring system had a much better predictive value, as shown in Table 4. Furthermore, as shown in Figure 6, the ROC curves also showed that the AUC for other single independent predictors (largest AUC = 0.761) had a lower discrimination power than the scoring system (AUC = 0.795).

**Table 3:** Scoring system for predicting the prevalence of radioiodine refractory+ status

Variable	Odds ratio	Score
Smoking	2.304	2
Type of tumor (FTC)	8.104	8
Extrathyroid extension	2.403	2
Lymph node metastasis number (≥4/<4)	3.399	3
Lymph node metastasis rate (≥53%/<53%)	6.418	6
pN stage (N0 or N1a/N1b)	3.185	3
		Total: 24

FTC, follicular thyroid cancer; p: pathological.

**Table 4:** Predictive value for the scoring system and independent risk factors

Variable	Sensitivity (%)	Specificity (%)	Youden index
Smoking	24.1	89.3	0.134
Tumor type (FTC)	8	98.7	0.067
Extrathyroid extension	29.5	84.8	0.143
Lymph node metastasis number (≥4/<4)	78.6	67.9	0.465
Lymph node metastasis rate (≥53%/<53%)	69.6	82.6	0.522
pN stage (N0 or N1a/N1b)	50	80.4	0.304
The scoring system	77.7	81.2	0.589

FTC, follicular thyroid cancer; p, pathological.

## 4 DISCUSSION

Differentiated thyroid carcinomas are often curable with surgical resection and ^131^I ablation because ^131^I can effectively treat tumor foci exhibiting high ^131^I uptake.^12^ In a few patients with metastatic disease, this capacity is lost due to tumor cell dedifferentiation. RAI refractoriness is uncommon, and patients may survive in the absence of treatment for years or decades with a stable or slowly progressive disease. However, many patients may require treatment when the tumor burden is large and when progression has been documented.^13^ According to the literature, in patients with localized DTC, the 5‐ and 10‐year relative survival rates reach 95% and 86%, respectively.^14^ In contrast, patients with the loss or absence of RAI uptake show 5‐ and 10‐year relative survival rates as low as 50% and 34%, respectively.^15^ Previous studies have shown that an older age (≥60 years) and the male gender were independently related to cancer‐specific survival in RAIR patients.^16^ In Johanna's study, progressive disease and a period from the initial DTC diagnosis to the diagnosis of RAIR cancer <3 years were the only independent prognostic factors for poor overall survival and carcinoma‐related death.^17^ Meanwhile, numerous studies have investigated mechanisms underlying the process of dedifferentiation and explored various therapeutic strategies to improve iodide uptake.^2^, ^3^, ^10^ Iodide trapping is a thyrotropin‐regulated mechanism involving an energy‐dependent transport mediated by the sodium/iodine symporter (NIS) at the basolateral surface of the thyrocyte and passive transport at the apical surface.^18^ In Riesco's and Knauf's study, the oncogene BRAF^V600E^ is associated with dedifferentiation due to the impairment of Na+/I‐targeting to the membrane^7^ and RET/PTC‐induced dedifferentiation of thyroid cells is mediated through Y1062 signaling through SHC‐RAS‐MAP kinase.^19^ Souza reported that P13K/Akt mTOR can downregulate iodide uptake in thyrocytes through downregulation transcriptional level of NIS mRNA.^20^ In the matter of epigenetic inheritance, the phenomenon that hypermethylation of TSHR, NIS gene promoters,^21^ and histone acetylation^22^ will decrease the effectiveness of radioactive iodine therapy was discovered by Smith and Zarnegar. In recent years, multiple strategies have been investigated for their potential to induce redifferentiation of TC cells, with limited success for nonspecific modalities such as retinoic acid,^3^ inhibitors of mammalian target of mTOR^10^ and histone modification agents.^22^ A much higher therapeutic efficacy was reached by treatment with (combinations of) specific oncogene‐guided kinase inhibitors, including MAPK, MAPK kinase, mammalian target of rapamycin (mTOR), and Akt kinases. Currently, MKIs represent the first‐line treatment for advanced refractory DTCs. Although there is no demonstrated benefit regarding overall survival and the quality of life is altered during treatment, a phase 3 study demonstrated a significant improvement in progression‐free survival (PFS) with MKIs over a placebo.^23^, ^24^ Better knowledge of the natural history of RAIR cancer is crucial when accurately selecting potential candidates and optimizing patient management.

This study showed that the prevalence of RAIR was 2.2%, which was in accordance with another study^25^ showing that the occurrence of RAIR cancer was 2.1% in females and comparable among males, at an incidence rate of 2.4%. Our data demonstrated that recurrence was decreased and survival was increased significantly for non‐RAIR patients compared with RAIR patients. Using univariate analysis, we found that twelve predicting factors were significantly associated with the RAIR status. Although the average largest diameter of metastasized lymph nodes in RAIR group and control group did not show the differences in initial data, our center has found the largest diameter of metastasized lymph nodes makes contribution to recurrence.^26^ In addition, six independent predictors of RAIR cancer, smoking, tumor type, extrathyroid extension, lymph node metastasis number, lymph node metastasis rate, and pN stage were confirmed by multivariate logistic regression analysis. According to the ORs, characteristics that were positively correlated with the occurrence of RAIR cancer were assigned different points, and a 24‐point scoring system was established. A cutoff value of 7 was found to be the best score for predicting the prevalence of RAIR cancer, with an AUC of 0.876. The sensitivity, specificity, and Youden index of this scoring system were 77.7%, 81.2%, and 0.589, respectively. The scoring system had a higher discrimination power than other single independent predictors in the current study.

To the best of our knowledge, the present study may be a novel report identifying the independent risk factors associated with RAIR cancer and establishing a scoring system to predict the prevalence of RAIR cancer in DTC patients. However, there are still several potential limitations to this study. First, because this was a retrospective review, the data were retrospectively collected and analyzed. The selection of control group may generate a selection bias although we have tried to minimize the comparison bias and our study analyzed data from a limited follow‐up period (16‐68 months; median, 32 months); thus, disease recurrence and disease‐free survival may be underestimated and overestimated, respectively. Second, this study mainly followed a cross‐sectional design, rather than using longitudinal observations, and single‐center verification may not be sufficiently accurate. Third, the scoring system in the current study was not validated due to limited positive cases. Multicenter and large‐scale validation to verify the predictive value of the system is needed to determine whether the characteristics of this study population were involved in generating these observational results.

## 5 CONCLUSION

The predictive model based on 6 factors provides a simple tool for predicting the development of RAIR cancer and showed excellent discrimination power. Patients with an index score of ≥7 points could be considered at a high risk for the postoperative RAIR cancer, and active surveillance or aggressive treatment may be recommended in subsequent therapy.
